# Typhoid Fever1Read at April meeting of the Roswell Park Medical Club.

**Published:** 1904-05

**Authors:** Thomas Bagley

**Affiliations:** Buffalo, N. Y.


### Typhoid Fever.[1]

By THOMAS BAGLEY, M. D., Buffalo, N. Y.

TYPHOID fever is a specific infectious febrile disorder, sporadic or epidemic, often communicated by the contagion from the stools, and characterised by lesions, chiefly of the intestines, mesenteric glands, and the spleen, with the presence of the bacilli of Eberth. The disease is marked, clinically, by a variable febrile course continuing, as a rule, three or four weeks, by a rose colored macular eruption, and by pulmonary and abdominal symptoms. Of the 100 or more names applied to this disease, the one seeming most appropriate was given by Louis in 1829—namely, typhoid, meaning stupor from fever. The disease is supposed to have been known to Hippocrates, the Father of Medicine, in 460 B. C. Galen mentioned it in his medical works in 130 A. D., as hemetritious. It was not until the seventeenth century that Spigelius discovered the intestinal lesions. The distinction between typhus and typhoid was made clear by Gerhard and Pennock, of Philadelphia, in 1837, thus giving the true nature

1. Read at April meeting of the Roswell Park Medical Club.

of the disease to America before Jenner made it known to England or France.

Typhoid is one of the most widely distributed infectious diseases. It occurs in all climates, but mostly in the temperate zone. It is most frequent in the late summer or autumn, coming after a dry, hot season. It is no respecter of sex; it may occur at any age, but more frequently between the fifteenth and twenty-fifth years, less often after the thirty-fifth year, occasionally in old age, and sometimes in the fetus or infant. Some persons are more susceptible to the poison than others, a catarrhal condition furnishing a favorable soil for it.

The exciting cause is the bacillus of Eberth. It is a small bacillus, about one-third the diameter of a red-blood corpuscle, rounded at one or both ends. In the center may be seen a round shining body, either a spore or a degenerative alteration in the protoplasm. These bacilli occur singly or joined together, end to end, in filaments. They are formed chiefly in the spleen, intestinal glands, mesenteric glands, in the liver, the kidneys, the urine, the sputum, the blood taken from the red colored spots, the brain, the spinal cord, the lungs, the testicles, the heart, and in the pus taken from the pleural cavity. They have also been found in the placenta of a woman in the fourth month of pregnancy on the twelfth day after an attack of typhoid, and occasionally in the liver, spleen, and blood of the fetus. It is thought that the bacilli cannot be transmitted from the mother to the fetus unless there has been an injury to the placenta. Dr. Pfeiffer was the first to discover them in the stools. They are rarely detected before the period of ulceration, when they become much more numerous. They disappear, as a rule, about the twenty-second day.

The bacilli produce pure cultures on potatoes, gelatine, agar, blood serum, and bouillon. They grow rapidly in sterilised milk. They have been known to live in milk thirty-five days, and in butter twenty-one days. Almost all articles of diet form a good culture medium for them. Many of the cultures, however, are not characteristic, for they bear a resemblance to the colon bacillus, for which they are often mistaken. Some investigators think the Eberth bacillus is not a specific microörganism, but a modified form of a number of other closely related germs. They have been known to live in the human body for a period of fifteen months after convalescence. Twenty minutes exposure to moist heat kills them. Heavy frost fails to destroy them. They retain their vitality in ice for months. Sunlight is disastrous to their growth. They usually enter the system by the intestinal mucous membrane, while some think at times by the respiratory tract.

They grow at the expense of the tissues, and develop certain toxic agents, which produce vomiting, purging, and rise of temperature.

Typhoid is not considered contagious in the ordinary use of the term. There are no exhalations from the lungs or skin which impart the disease. It is infectious, the poison being found chiefly in the stools. However, persons handling the soiled linen of typhoid patients have contracted the disease. It is generally admitted that the dry discharge from a patient floated upon the air and breathed into the lungs of another may carry it. The lesions of typhoid are divided into two groups: first, those characteristic of the disease; second, those regarded as secondary changes, due to the effect upon the tissues of the constitutional infection, and the long continued fever.

The characteristic post-mortem changes are seen in the lymphatic structure of the intestines, in the mesenteric and other glands, and in the spleen. The alterations taking place in these glands are divided into four stages: (1) infiltration, (2) necrosis, (3) ulceration, and (4) cicaterisation. These stages may exist in different glands at different times. The neighborhood of the ileocecal valve exhibits the most advanced stages of the glandular lesions. The enlargement of the spleen begins in the middle of the first week. It may be three times its normal size, and it may return to its normal size at the end of the fifth week.

The period of incubation is thought to be about two weeks, though it is sometimes less. Cases of from one to four days duration have been reported. A sense of weakness and fatigue from exertion, light and disturbed sleep, confusion of ideas, loss of appetite, colicky pains, diarrhea, nausea, coated tongue, epistaxis, headache, aching limbs, dulness of hearing, and rise of temperature toward evening, are premonitory symptoms. The appearance during the first week is listless and apathetic. Delirium often occurs during the night. The pulse is increased, often full and of low tension, but later dicrotic.

The abdomen may become disturbed. Tenderness and gurgling sounds may be found in the left iliac fossa. At first there may be constipation; later, diarrhea with the characteristic pea soup stools. Rose colored spots appear about the seventh to the tenth day. The urine is often scant and feverish, and may contain albumin. During the second week the symptoms become aggravated. The temperature may become remittent during the third week. These symptoms may persist until the fourth week. The blood shows but little change until the third week, when the number of red corpuscles and the percentage of hemoglobin become lessened. The number of leukocytes is not very much affected. Dry tongue, sordes on the teeth, stupor, rapid pulse, urine and feces passed unconsciously, great emaciation, and bed sores—all these denote what is termed the typhoid state. If the patient lives, these symptoms gradually subside and convalescence begins.

Typhoid fever is usually followed by one or more complications, perhaps the most serious of which is perforation of the intestine. It occurs in about 2 or 3 per cent. of all cases and is a common cause of death. It is usually looked for during the second and third week, but has occurred as early as the eighth day. It has been known to happen after the patient has fully recovered and returned to accustomed employment. Peritonitis, local or general, often follows perforation. Venous thrombosis is a frequent complication, often occurring in the femoral vein, producing edema and pain. It is more apt to affect the left leg as the left iliac is crossed and pressed upon by the right iliac artery. Sudden death is sometimes due to the transit of a thrombus from the limb to the heart, and septicemia may occur through the softening of the clot. Death also may occur simply from exhaustion after the fever has left. Falling of the hair is common after this disease. Herpes labialis, pericarditis, endocarditis, myocarditis, valvular troubles, anemia, rupture of the spleen, necrosis of the nose cartilage, edema of the glottis, perichondritis, bronchitis, lobar pneumonia, pulmonary edema, pleurisy, and acute miliary tuberculosis are other complications that may follow typhoid. Laparatomy has been successfully performed in 15 out of 2,000 cases of this disease.

Relapses are apt to occur in the second or third week of convalescence and after the absence of fever for several days. The average duration of this disease is from three to four weeks. It may prove fatal by the fifth or sixth day, and, in the maligant type, even earlier. The third week is the time of greatest mortality. Brand's method of treatment has reduced the mortality in hospitals from 10 to 30 per cent. to 5 to 8 per cent., in private practice to 10 per cent.

The prognosis is difficult. It is less fatal in children from infancy to puberty. It increases rapidly after the forty-fifth year. Some claim that the robust and corpulent more readily succumb to it; in the latter the fever runs very high. For persons of intemperate habits, and with gouty or venereal affections the prognosis is unfavorable. In pregnant women the tendency is to abort, usually with fatal results. Early development of nervous symptoms and delirium is unfavorable. If the germs which produce toxemia can be removed the disease may be prevented or aborted. General prophylaxis should be observed. A large well-ventilated room is needed for the patient. Rest in bed is necessary from the first. The bowels and bladder need early attention. Liquid diet is safest even for a week or ten days after the fever has subsided. Alcohol should be avoided if possible. It has been said that using it indiscriminately is like borrowing money at 100 per cent.; it must be paid back at high interest. Antipyretics should be used cautiously. Strict antiseptic treatment is of the greatest importance. Hydrotherapy is a constant feature in the treatment of typhoid, and good nursing is indispensable.

1344 West Avenue.

DISCUSSION.

Dr. Geo. F. Cott.—Very common lesions of the throat and larynx in typhoid are catarrhal ulcerations and paralyses. The reason why we do not have very accurate statistics with regard to these conditions, is because these parts are not examined unless there is some complaint made by the patient. In the mild types of the disease they are overlooked, often also in the severe forms, even in case of death. Lesions are noted clinically in only about 3 per cent. They are found post-mortem in 17 per cent., always of a severe nature. Some lesions are almost always due to perichondritis. The diphtheritic variety is always secondary. The bacilli have been searched for unsuccessfully. When we have severe lesions like perichondritis there is superficial inflammation always following. The cricoid cartilage has been found affected in 80 per cent. of the cases. In that it differs widely from other diseases like syphilis and tuberculosis, in which it is rarely affected. In these diseases the arytenoids and epiglottis are more commonly invaded.

Typhoid is differently considered in the old country. There they call it typhus, and divide it into abdominal typhus and petechial or exanthematous typhus. Cases of deep ulceration are usually found in the exanthematous variety, and are called laryngotyphus. Stenosis and edema are complications that may occur from the involvement of neighboring structures, especially the thyroid gland, by which pressure causes stenosis of the trachea and larynx. The edema is supposed to be due to nearby lesions invading the larynx and causing perichondritis. Paralysis of the laryngeal muscles is a post-typhoid condition, which occurs about the fourth or fifth week. The observer who has made the best report to date, Przedborski, had 25 out of 100 cases of typhoid of the abdominal type. He had 7 out of 25 cases of the exanthematous form, all of which recovered.

The throat and larynx are most commonly affected by catarrhal ulcers and also deep ulcerations. The nose is comparatively immune to this disease; rarely catarrhal ulcerations of the cartilaginous septum produce perforation. One very common lesion, from which diagnosis is often materially assisted, is epistaxis which occurs from the septum. Occasionally, the patient will cough up blood, which is almost invariably dark in color, because it has trickled from the nose into the throat. One also finds a condition known as rhagades, consisting of little cracks or fissures which bleed readily, and are noted almost always in the pretyphoid condition.

Superficial ulcerations which epithelium breaks down, leaving a grayish-yellow base, always get well, unless the patient has had some other severe conditions mentioned and which are very uncommon, as perichondritis and the like. They do not leave a scar, and they get well without treatment. When there are deep ulcerations, even if they do get well, they leave more or less cicatricial contraction as in syphilis. I know of a patient who had such a typhoid ulceration in the soft palate, a part seldom affected by ulceration or paralysis, in whom one quarter of it was destroyed. These lesions may occur in any period of the disease, and they may always be sought. They may produce very serious consequences in severe types. They should be treated with antiseptics. Deep ulcerations are supposed to be produced by mixed infections. In many cases the typhoid bacillus cannot be found. They are caused primarily by the debilitated condition of the patient, which gives opportunity for the inroads of mixed infections.

Epinger considers ulcers of the mucous membrane of the throat and larynx analogous to lesions in the intestines, and that whenever there is adenoid tissue, infiltration takes place and it breaks down. These typhoid ulcers are very characteristic in location, being found on the pillars of the fauces and on the laryngeal border of the epiglottis; in the aryepiglottic folds; seldom, yet occasionally, in the subglottis; very rarely if ever on the vocal chords. A condition has been described as diphtheritic typhoid in which a diagnosis of diphtheria and typhoid has been made. It is considered to be due to deep ulceration with membrane formed over them. Another condition worthy of note, where there is no particular lesion is dry mouth, the tongue being dry and hard. The temperature cannot be gotten in the mouth, or appears subnormal, while quite apparent if taken per rectum.

Ear complications are usually late, practically post-typhoid, seldom occurring before the fourth week, and from that time on, indefinitely; occasionally where there has been chronic suppuration of the middle-ear, when the temperature begins to rise, the suppuration ceases; as soon as the fever subsides the discharge begins again. The aural conditions are simply inflammatory, giving rise to more or less pain, suppuration with perforation, and profuse discharge of pus which is due to mixed infection. It may get well without any special treatment.

The next severe condition is inflammation of the mastoid process. That very often occurs before one notices any trouble with the ear except pain. It apparently may arise simultaneously with the inflammation in the ear, before perforation of the drum. Bezold found in nineteen cases out of forty-one, marked tenderness over the region of the mastoid antrum, about the level of the upper border of the external meatus and a little behind it. In the mastoid symptoms occurred coincidently with the inflammation. In five out of forty-one there were periosteal abscesses over the mastoid without destruction of the bone. Only one case is on record where an aural inflammation due to typhoid produced thrombosis of the lateral sinus and, secondarily, death. The parotid gland is subject to inflammation in a certain number of cases. When it suppurates, it is very likely to discharge into the external canal and should not be mistaken for middle-ear infection. The reason is, there are little fissures in the cartilage, generally three, which are readily punctured by the pus, and the parotid lies immediately under the cartilage. Deafness results in the vast majority of ear complications. Why? No satisfactory explanation has been made.

Dr. J. C. Thompson.—Historically speaking, typhoid is one of the oldest diseases of which we have any record in the history of medicine. In the temperate and torrid zones, it has been one of the most important afflictions incident to humanity. In the dark ages, when little was written on medical subjects, many of the epidemics ascribed to plague and typhus fever, were in reality outbreaks of typhoid; at least such now is believed to be the fact. It was not until the early part of the past century, that any differentiation of the latter diseases was attempted, and to the American profession belongs the honor of the achievement, won by two Philadelphia physicians, Gebhardt and Pennock. English and continental workers soon verified their position. But in England, Budd gave the world the largest amount of information as to the real characteristics of the disease, its etiology, symptomatology and clinical aspect, work which has placed him in the front rank among all the students of this subject up to the present time. He demonstrated its infectious nature, that the agent existed in the stools, and that it gained access to the organism through the mouth. Murcheson, also an Englishman, contributed largely to the study of this disease. Coming down to the period of the germ theory, Eberth's discovery demonstrated clearly the infectious microörganism. The vitality of the bacillus is astounding. It has been found to be viable in gallstones after a period of twelve years. In fact, their vitality seems to be almost indefinite when properly protected from sunlight and under otherwise favorable conditions.

Budd and Murcheson worked out the methods of transmission very clearly, the latter stating that it was obtained through food, water, and the like, although it will be remembered that the infecting agent was not to be discovered until many years afterward. The question of infection through the lungs was also considered by the two men, a point on which there has been considerable difference of opinion at the present day. Wasdin has evolved a theory, and demonstrated its truth to his own satisfaction, as to the pulmonary invasion of the disease. In a recent paper, which I was so fortunate as to hear, he stated that the so-called cycle of twenty-one days is a mistake; that it is a cycle of fourteen days; that the first infection is by way of inspired air, because he has been able to demonstrate the presence of the bacillus in the sputum before it can be found in the stools; that, therefore, the infection of the alimentary tract is secondary to the lung infection, requiring about seven days to reach Peyer's patches after the primary infection of the respiratory tract. He bases his opinion on the fact that he has been able to isolate the bacilli from the sputum, not from the buccal secretion, but from the trachea and lungs, for a week before they appear in the discharges from the bowels. He believes that the cycle is fourteen days; and that when it runs beyond that period, it is due to a reinfection of the intestinal tract from the lungs, which gives it another cycle of fourteen days. He is still working along this line with much promise of effectually establishing his position. His observation was the first to be recorded in the city on the treatment by acetozone at the marine hospital in 1901. He believes it inhabits the growth of all organisms in the intestinal tract. After its use, he found absolutely sterile stools. Many of his cases recovered after a period of fourteen days instead of twenty-one days. They may be autoinfected at the end of a week, causing another run of fourteen days, which terminates both cycles at twenty-one days. They may be reinfected at the end of the second week, causing a double period of fourteen days, or four weeks.

In regard to the symptomatology it is hardly necessary to speak. It was the two physicians before referred to, Gebhardt and Pennock, who pointed out the difference between the rose-colored spots of typhoid and the petechial eruptions of typhus. The latter was an extremely common disease at the time. As a rule, it is a typical case we are called upon to treat in private practice, modified by circumstances and individuality of patient. Aids to the diagnosis are the diazo reaction of the urine, Widal for the blood and, of course, the pathognomonic sign, the bacilli in the dejecta, aids which are too often beyond the reach of the less fortunately situated practitioner. He has to depend upon the sum of his general experience and that sixth sense which becomes greatly developed by long practice. In diet, milk is a sheet anchor. Broths may be depended upon a great deal.

There are one or two things in regard to treatment worthy of special mention. One is that most of the annoying symptoms result, not only from the intoxication resulting from the bacillus of the disease, but also from activities of other organisms, such as the streptococcus, the colon bacillus, and the like, which increase with great rapidity owing to the lowered vitality of the body.

One thing I depend upon is careful attention to the mouth, keeping it constantly rinsed with some good antiseptic solution. I believe that it helps to prevent the development of various germs which find, in decaying parts of food retained in the mouth, a ready nucleus for their luxuriant growth. I also believe in the use of strychnine in this disease. Subcutaneously in fairly large doses, it acts as a true tonic to the nervous system, and if you can tone that up to a high state of efficiency, you will prevent much of the toxemia that would otherwise supervene. I usually begin with 1-30 grain twice a day and increase as indications demand, beginning early in the disease as I would also in the pneumonia of adults. The chaotic state of opinion as to the treatment of typhoid is evidence of indecision with regard to it. As a matter of fact, the patient will not infrequently recover if left alone. I believe too much treatment does much harm: that we can do more by hygiene and general management than by drugs.

Dr. J. H. Potter.--In presenting the surgical aspect of typhoid, I will refer first to the vitality of the germs. Curiously enough they have been found in abscesses about the gallbladder fourteen and one-half years after recovery from the fever, in bone lesions from four to five months and as late as six years. There is hardly any organ in the body in which they may not be found. Nearly all pathogenic organisms have been found associated with the typhoid bacillus. In some lesions following the disease pure cultures of Eberth's bacillus have been found; in others, none at all. Where they are not found, the characteristics of the local condition are those which attend the activities of the particular germ present. In eighty-four test cases pure cultures were found in sixty-three; the remaining number were either mixed or contained none. This fact also establishes the pathogenic character of the bacillus. At one time it was claimed that the typhoid bacillus could not be classed as a pathogenic organism.

Among the more important surgical aspects of the disease are perforations, lesions of the gallbladder and liver, of bones, abscesses, joint infections, gangrene, and the like. Perhaps the most important is intestinal perforation. It is not necessary to discuss its pathology, for we all know why it occurs. It has been estimated that perforation accounts for about 6 per cent. of the mortality due to typhoid fever. About 71 per cent. of all perforations occur in males. In childhood it is quite rare. It is most frequently seen in about the third or fourth week, but has been recorded anywhere from the first to the sixteenth week. Its most frequent site is in the ileum, next in the colon, and it has been found in the appendix. As a rule, there is only one, but several have been found. Death results, usually, in from one to seven days. The severity of the disease bears no relation to the probability of this accident, as it may occur in the very mildest cases.

The diagnosis is sometimes attended with difficulty. In eighty-six cases, fifty-six had a sudden onset; in fifteen it was gradual; in five there were no symptoms whatever, the lesion being discovered post-mortem. The usual symptoms are those of shock, sudden pain, vomiting, occasionally accompanied by a marked fall in temperature. One peculiarity of the condition is that the hepatic dulness is not lost, as one might expect it to be from the escape of gas in the bowels. One point which assists diagnosis is the usual absence of marked leucocytosis in typhoid fever; if it rapidly increases it is probable, other symptoms corroborating, there has been a perforation. Death due to septic peritonitis is the usual sequence, the average death-rate in perforations being 95 per cent. When the diagnosis is reasonably certain and the condition of the patient justifies it, operation should be advised. The time selected is of the greatest importance. As a rule, it is better to wait until the patient has rallied from the shock, probably about the second twelve hours. The percentage of recoveries in operative cases is quite fair, the death-rate being reduced from 95 to about 80 per cent.

Another complication which may be regarded as surgical is hepatic abscess. It is a somewhat rare, as well as interesting condition, several unsuspected cases having been discovered post-mortem. Unfortunately, if suspected, the treatment must be largely symptomatic as surgical intervention does not hold out much prospect of recovery. In contradistinction to hepatic abscess, abscess of the gallbladder is comparatively frequent. In at least half the cases of trouble with the gallbladder, the symptoms are not distinctive. When there is pain in the region of the gallbladder, and it is evidently distended, the question of opening it should be seriously considered. When we are reasonably sure that it has been perforated, surgical interference should be instituted at once. The prognosis in these cases is better than in perforation of the intestines, for the reason that the contents are not so toxic as those of the intestines.

The next point for consideration is bone lesions, due either to the typhoid bacillus or the mixed infection, usually the former. These lesions, as a rule, are quite late in their appearance, sometimes requiring months, or even years for their development. It is supposed that some blow or local injury lowering the vitality of the part, affords opportunity for the development of the infection already deposited there. As to the character of lesions, they are varied, such as necrosis, caries, bone abscess, osteomyelitis, exostosis, and the like; in fact, nearly all varieties of the bone lesions may follow. They occur more frequently in males than in females. Practically, any bone of the body may be involved, though those of the lower extremity are the most frequent seat of injury. Next come those of the trunk, especially the ribs, then the upper extremity, and lastly, the head. The symptoms are usually the local ones which you get from mixed infections. The constitutional symptoms are slight in bone lesions due to typhoid bacillus; there may be some slight tenderness or pain, some thickening, and the like, and if not relieved surgically the formation of a sinus of sluggish characteristics may occur. If the infection be mixed, the constitutional symptoms are more marked, and the prognosis more serious accordingly. The treatment of these cases is almost entirely surgical though details are not necessary to enter into here.

Post-typhoid abscesses may occur in almost any organ of the body, and they may be single or multiple. If multiple, the case assumes more of the pyemic type. The infection may be pure or mixed. The symptoms in all such cases are those of abscess formation in the particular part which may be under consideration. The treatment may be surgical. In an abscess of slow development it should be opened under the strictest precaution, lest we add mixed infection to the trouble already existing. Gangrene may follow typhoid fever. It is quite rare, however, and is a late complication. It occurs any time from the fourteenth day to the seventh week. It is caused by either a thrombus at the seat of the trouble or by an embolus. It may be produced by infection attacking the vessel-walls. If the obstruction occurs in an artery, it causes dry gangrene; if in a vein, the moist variety. This condition develops more frequently in males than in females and most frequently in the lower extremities. It also has been found about the nose, ear and trunk.

The treatment of threatened gangrene resolves itself into efforts to promote and maintain circulation by toning up the heart, alternating heat and cold, moderate friction, stimulating liniments, alternating electric current; in short, anything to keep up the blood supply to the threatened area. If the onset cannot be prevented, suitable antiseptic dressings must be applied and the dead tissue removed; if in an extremity we must await the line of demarcation and then operate. Infection of joints may be single or multiple and usually occurs during the period of convalescence. When the inflammation is subacute if the joint be kept at rest, the condition will often subside. However, if this subacute inflammation progresses and the joint becomes filled with fluid, the ligaments may become elongated and give rise to a luxation. Comparatively speaking, it is quite a common occurrence suddenly to find a dislocation of the hip, and unless recognised serious consequences may fellow. As a general thing, especially about the hip, the symptoms are not marked. The patient is often more or less apathetic during the period of the disease, so that the joint condition may go on until by some movement you get a dislocation which remains unrecognised for a considerable time. These joint affections occur more commonly under the age of thirty.

As to treatment, if we find a joint filled with fluid which shows no tendency to subside, it should be aspirated and, if pus be found, opened and drained. If it be a pure typhoid infection, the prognosis is quite good. We rarely find hematomas, though they have been found in the abdominal walls. This affection occurs late in the disease and the treatment is surgical. All cases operated upon have recovered. All that have not been operated upon have died. They should be evacuated and drained. Cerebral complications are brain abscess, meningitis, and thrombus. Cerebral abscess following typhoid is almost invariably fatal. When diagnosis is possible, it is perfectly justifiable to trephine. Meningitis is difficult to diagnosticate because the mental state of the patient, due to the primary disease, is such as to mask the secondary condition. Empyema of lung should be treated surgically, and the same advice applies to purulent pericarditis.

Dr. A. G. Bennett.—There is practically no literature on typhoid effects on the eye. Personally, I have knowledge of only three cases. Many patients have consulted me in what may be called a pretyphoid state, complaining of headache and distress in the eyes, symptoms that later passed away. We also frequently get patients who, after an attack of typhoid, complain of discomfort in the use of their eyes, while they never had trouble with them before. One can hardly refer to it as a post-typhoid condition, because the same may be seen after any debilitating illness, for instance after parturition. Many women complain of their eyes during the lying-in period. It is a result of a weakened condition which may bring some latent refraction or muscular defect to the surface. It is doubtless due to the peculiar fact that the loss of weight does interfere to a certain extent with the curvature of the cornea; the amount of fat in the orbit being decreased, there is less pressure exerted from behind and a change in the curvature of the cornea results. We may find astigmatism or hypermetropia, which are corrected by regaining the normal weight.

The conditions which may be ascribed directly to the typhoid state are few. The most serious is inflammation of the optic nerve, probably to be followed by atrophy and permanent blindness. Of course, every optic neuritis is not followed by blindness, but it is very liable to occur. I have had one case following typhoid neuritis. Within the past year I have seen a patient several times, in whom there was an embolus in a fine twig of the retinal artery. The area supplied by this twig was totally blind. You can readily understand what would have happened had the embolus lodged in the main artery.

A most interesting typhoid complication was the condition of Chester Cott. It began as a conjunctivitis and gradually invaded the corneal layers, but never got very deep. I believe if we had made a culture we should have found the typhoid bacillus. The cornea was locked in the epithelial layer only and produced a number of ulcerations. So far as my experience goes, it is a unique case. It appears very much like herpetic eruptions of the cornea, but not so painful. I believe it will clear up without leaving any marks at all, because it is so superficial. So far as the treatment of the condition is concerned, it depends upon the state of the patient. In neuritis, strychnine would be indicated. I doubt whether the oculist would have more to do than to make a diagnosis; there is practically nothing to do in atrophy.

Dr. F. C. Busch.—I am expected to speak of the stomach in typhoid fever. I suppose that means during and after typhoid. In this disease, the stomach is less liable to have anything the matter with it than any other organ in the body, because the period of disease is a period of rest for the stomach. There are a few gastric complications which rarely occur in typhoid; nausea and vomiting are the most common, and may occur early or late. When early, the cause is rather hard to state, unless it be a reflex result of the toxemia. When it occurs late it is more likely to be due to dietetic errors, irritating medicine, or the result of peritonitis following perforation. Most rarely it is due to typhoid ulcer in the stomach itself.

During the fever there may be a diminution in, or complete absence of, secretion of hydrochloric acid. Investigations on this subject have led to various results. It was found by one observer that diminution or complete absence occurred in nearly all his cases. Another found that free hydrochloric acid was present during continuous fever and transitory high temperature. Others state that, as a rule, the secretion of pepsin is not affected. However, all these results may be found in any continuous fever and are not the result of typhoid per se. As a sequela there may be chronic gastric catarrh with dilatation, a result, too, of other debilitating infectious diseases. The dilatation is due to loss of tone and motor power, as a result of the general weakness and toxemia. Then, typhoid convalescents have a ravenous appetite and when allowed to eat without supervision, are likely to eat to excess, thus inducing this condition of chronic catarrh and dilatation.

Dr. J. A. Gibson.—The part that the nervous system plays in this disease may be intimated by the fact that the Germans call it "nervenfieber." The most common symptoms in connection with the nervous system are headache and insomnia. While the headache is one of the earliest symptoms, the insomnia is the most troublesome. Paralysis of the higher centers occurs, although not very frequently. Delirium is another common symptom and it is generally most marked at night. It may be of different varieties: quiet, in which the patient knows that he has delusions, but cannot help seeing them, though aware all the time that the phenomena are unreal; they may be violent or maniacal, and it is to be remembered that suicidal tendencies are quite common in severe types; in alcoholics, delirium tremens is a common complication, usually occurring about the second week. In females we see a certain delusion of presentation, which is quite common.

With regard to diagnosis, the delirium may be confounded with that due to meningitis, a disease hard to differentiate, and, as one of the previous speakers remarked, we often find, on postmortem, that there may have been a meningitis also. In children, convulsions are another complication. I came across one authority, who mentions that the knee reflexes are frequently exaggerated in this disease, which does not indicate anything in particular. He also stated that where emaciation was very marked, you might get idiopathic muscular contractures. Of sequelæ several have been mentioned by previous speakers. Mental disease, loss of memory, impairment of intellectual faculties are noted. Aphasia occurs, as do serious cases of neurasthenia. These may be cerebral, cerebrospinal, visual, or sympathetic.

A number of localised pains are liable to follow typhoid, particularly in the soles of the feet and toes, slight pressure causing great pain. It is probably due to a local neuritis from tissue poverty and faulty nutrition. Disseminated sclerosis is said to occur quite frequently, as well as poliomyelitis. I saw one case where the symptoms closely resembled those of bulbar paralysis. The patient had difficulty in speaking, and in executing the finer movements of the fingers. It terminated fatally. Paralysis of the cranial nerves is rare. Hearing is commonly affected, as explained by Dr. Cott. Peripheral neuritis has been noted quite frequently.

Dr. THOMPSON inquired as to the cause of the head symptoms. Dr. Gibson stated that the bacilli had been found in the cerebrospinal fluids; that they are probably due to a localised meningitis.

Dr. DIGNEN suggested the thought, in connection with the discussions of Drs. Thompson and Potter, that possibly the body contained supplies of the bacillus all the time and required only a suitable opportunity to begin its activities.

